# Therapeutic potential of brain stimulation techniques in the treatment of mental, psychiatric, and cognitive disorders

**DOI:** 10.1111/cns.13971

**Published:** 2022-10-13

**Authors:** Jose Antonio Camacho‐Conde, Maria del Rosario Gonzalez‐Bermudez, Marta Carretero‐Rey, Zafar U. Khan

**Affiliations:** ^1^ Laboratory of Neurobiology, CIMES University of Malaga, Campus Teatinos s/n Malaga Spain; ^2^ Department of Medicine, Faculty of Medicine University of Malaga, Campus Teatinos s/n Malaga Spain; ^3^ CIBERNED Institute of Health Carlos III Madrid Spain

**Keywords:** closed‐circuit neuromodulation, deep brain stimulation, invasive brain stimulation, noninvasive brain stimulation, repetitive transcranial magnetic stimulation, transcranial direct current stimulation, transcranial magnetic stimulation

## Abstract

Treatment for brain diseases has been disappointing because available medications have failed to produce clinical response across all the patients. Many patients either do not respond or show partial and inconsistent effect, and even in patients who respond to the medications have high relapse rates. Brain stimulation has been seen as an alternative and effective remedy. As a result, brain stimulation has become one of the most valuable therapeutic tools for combating against brain diseases. In last decade, studies with the application of brain stimulation techniques not only have grown exponentially but also have expanded to wide range of brain disorders. Brain stimulation involves passing electric currents into the cortical and subcortical area brain cells with the use of noninvasive as well as invasive methods to amend brain functions. Over time, technological advancements have evolved into the development of precise devices; however, at present, most used noninvasive techniques are repetitive transcranial magnetic stimulation (rTMS) and transcranial direct current stimulation (tDCS), whereas the most common invasive technique is deep brain stimulation (DBS). In the current review, we will provide an overview of the potential of noninvasive (rTMS and tDCS) and invasive (DBS) brain stimulation techniques focusing on the treatment of mental, psychiatric, and cognitive disorders.

## Graphical abstract

Diagrams of representative non‐invasive and invasive brain stimulation techniques. (A) represents the standard figure‐eight transcranial magnetic stimulation (TMS) coil, which is connected to intensity and pulse regulator. (B) exhibits bipolar transcranial direct current stimulation (tDCS) electrodes and current regulator. (C) shows deep brain stimulation (DBS) microelectrodes inserted in brain and connected to a stimulator implanted elsewhere.

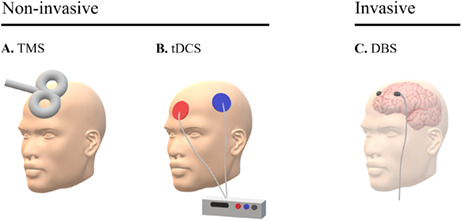

## 1 INTRODUCTION

Mental and psychiatric disorders are among the most prevalent illnesses worldwide. Nearly 50% of human population will meet criteria for a mental disorder diagnosis in their lifetime, more than 28% will meet criteria for an anxiety disorder, and more than 20% will meet criteria for a depressive disorder.^1^ According to the World Health Organization report, depressive disorders alone are the highest contributor to the burden of disease in middle‐ and high‐income countries and are the third highest contributor worldwide. Suicide, which is almost always associated with the presence of a mental disorder, is the third leading cause of death among persons aged 15–24 years, and fourth among persons aged 18–65 years. Available medications and other treatments are effective for many patients; however, in large number of patients, these methods have shown to be clinically ineffective. Therefore, in recent years, there has been increased interest in application of brain stimulation to circumvent this problem and to alleviate those patients from their suffering. Brain stimulation involves passing electric currents into the cortical and subcortical area brain cells to modulate brain functions and is performed by two methods: noninvasive and invasive. Technological advancements have contributed to the development of several techniques within both categories (see in Box 1). Noninvasive stimulation is performed with the use of two distinct techniques: transcranial magnetic stimulation (TMS), which was introduced in 1985,^2^ and transcranial electrical stimulation (tES), which has been in clinical use since 1801.^3^ Nevertheless, at present, the most used form of TMS is repetitive TMS (rTMS) and of tES is transcranial direct current stimulation (tDCS). Noninvasive brain stimulation techniques use electrical currents applied directly through magnetic fields or electrodes on the scalp of patients to stimulate cortical brain cells, whereas invasive stimulation, such as deep brain stimulation (DBS), involves passing electric currents into the subcortical area brain cells through surgically implanted electrodes deeper in the brain. Unlike invasive, noninvasive methods do not require anesthesia and surgical operation, and therefore, they are preferred over invasive methods. In the last decade, use of these brain stimulation methods has not only surged substantially but also has expanded to a wide variety of brain disorders, including schizophrenia, major depressive disorder, anxiety disorders, and cognitive dysfunctions. The effects of noninvasive repetitive TMS (rTMS) vary depending on the shape of the coil (figure of eight, H coil, double cone coil), pacing pattern (high frequency, low frequency, and theta‐burst), and stimulation site.^4^ The effects of tDCS also vary according to the type of current (direct, alternating, pulsed, and random noise), polarity (anodal or cathodal), current intensity, and stimulation site.^5^ Both noninvasive methods, rTMS and tDCS, have been used in clinical settings and are already regulated for clinical use in many countries, and currently, they are approved by the Food and Drug Administration (FDA).^6^, ^7^, ^8^ On the contrary, DBS is also an FDA‐approved treatment for patients with involuntary movement disorders since the late 1980s.^9^

Box 1 **Various types of brain stimulation**

**1. Noninvasive brain stimulation techniques** modulate brain excitability by the application of either magnetic fields over the head or electrical currents directly through electrodes placed on the scalp. There are several modalities of use in both the techniques.

1.1. Transcranial magnetic stimulation (TMS)

1.1.1 In **repetitive TMS (rTMS),** a figure‐of‐eight coil is used to stimulate precise but relatively superficial locations on the cortex through administration of short electromagnetic pulses.
1.1.2 In **deep TMS (dTMS)**, a H‐coil is used to target broader but deeper brain areas. However, similar to rTMS, short electromagnetic pulses are administered.
1.1.3 **Magnetic seizure therapy (MST)** involves the induction of a seizure by applying high‐intensity electromagnetic pulses. The stimulation is limited to a focused area in the brain, and therefore, it produces minimal effect in surrounding tissues. In contrast to rTMS and dTMS where patients are awake throughout the treatment, for MST, patient must be anesthetized.
1.1.4 **Stanford neuromodulation therapy (SNT)** is a form of accelerated magnetic pulse application, which is approved by the Food and Drug Administration as **Stanford Accelerated Intelligent Neuromodulation Therapy** (SAINT) for the treatment of depression.

1.2. Transcranial electrical stimulation (tES)

1.2.1 **Transcranial direct current stimulation (tDCS)** is the most modern and used version of tES. In tDCS, continuous but low‐intensity current is applied through electrodes (anode and cathode) placed on the scalp, whereas **high‐definition tDCS (HD‐tDCS)** is a variant of this technique and in contrast to tDCS where distribution of electrical current in a target area is relatively diffused, HD‐tDCS devices are used for increased focal stimulation of a target area.
1.2.2 **Cranial electrotherapy stimulation (CES*)*** is a form of neurostimulation that applies pulsed, low‐intensity current through electrodes placed on anatomical positions around the head, such as earlobes and temples.
1.2.3 **Transcranial random noise stimulation (tRNS)** is achieved by applying an alternating current which varies in frequency and amplitude (within a certain range) throughout the stimulation period. However, **transcranial alternating current stimulation (tACS)** is frequency specific stimulation, and therefore, current is applied at a fixed frequency rather than randomly acquired range of frequencies as in case of tRNS.
1.2.4 **Electroconvulsive therapy (ECT)** involves a brief electrical stimulation of the brain while the patient is under anesthesia. Electrodes are placed at specific sites on the scalp and electrical currents are passed through the brain to produce a brief seizure.

**2. Invasive brain stimulation techniques** generally involve surgery to implant an electrode deep in the brain to deliver electrical pulses at a high frequency. The intensity and frequency of electrical currents are controlled by a generator implanted under the skin of chest or head

2.1. **Deep brain stimulation (DBS)** involves application of continuous stimulation through a pair of electrodes implanted in a specific area of brain.

2.2. **Vagus nerve stimulation (VNS)** implicates the delivery of electrical pulses to the left vagus nerve through a device implanted under the skin.

2.3. **Closed‐loop neuromodulation (CLN)** is a stimulation that is automatically adjusted by an implanted deep brain sensing and stimulation device in response to changes in the patient's electrical brain activity. However, **Responsive neurostimulation (RNS)** is produced through a programmable CLN device, which releases small pulses or bursts of stimulation when detects beginning of seizure activity to stop the seizures in patients suffering with epilepsy.

## 2 NONINVASIVE BRAIN STIMULATION

### 2.1 Repetitive transcranial magnetic stimulation

rTMS is a neuromodulation technique that uses large transient magnetic fields to induce focal electrical currents in a specific brain area. rTMS induces short pulses of intracranial electrical currents and can produce changes in excitability of the cerebral cortex, locally as well as in neurons at areas far from the stimulation site, along functional anatomical connections,^10^, ^11^ release neurotransmitters,^12^ and induce synaptic plasticity^12^, ^13^ and metaplasticity.^14^ At the therapeutic level, rTMS is effective for the treatment of several mental, psychiatric, and cognitive disorders (see a summary in Table 1). In following, we describe on the therapeutic benefits of rTMS:

**TABLE 1:** Therapeutic benefits of application of rTMS in mental, psychiatric, and cognitive disorders

Disorder	Participant size	Stimulation site	Stimulus frequency	Outcome of treatment	Effect size or SMD and *p*‐value	References and comment
Major depressive disorder	1335	DLPFC	Low to high frequency (1–20 Hz)	Effective reduction in depression	2.35 (*p* < 0.025)	Leggett et al.^7^ (Meta‐analysis of 31 studies)
Anxiety disorder	98	DLPFC	Low to high frequency (1–20 Hz)	Improvement in anxiety symptoms	2.06 (*p* < 0.05)	Cirillo et al.^18^ (Meta‐analysis of 4 studies)
	164	DLPFC	Low to high frequency (1–20 Hz)	Improvement in anxiety symptoms	ND	Sagliano et al.^19^ (Systematic review 5 studies)
	152	DLPFC	Low to high frequency (1–20 Hz)	Improvement in anxiety symptoms	1.85 (*p* < 0.001)	Parikh et al.^21^ (Meta‐analysis of 6 studies)
	347	DLPFC	Low to high frequency (1–20 Hz)	Reduction in symptom severity	ND	Vicario et al.^20^ (Systematic review 21 studies)
Post‐traumatic stress disorder	421	DLPFC	Low frequency (1 Hz) High frequency (5–20 Hz)	Improvement in PTSD symptoms	0.70 (*p* < 0.05) 0.71 (*p* < 0.05)	McGirr et al.^29^ (Meta‐analysis of 10 studies)
	157	DLPFC	Low to high frequency (1–20 Hz)	Reduction in severity of PTSD symptoms	0.88 (*p* = 0.047)	Cirillo et al.^18^ (Meta‐analysis of 9 studies)
	377	DLPFC	Low frequency (1 Hz) High frequency (5–20 Hz)	Reduction in overall PTSD symptoms	0.92 (*p* < 0.05) 3.24 (*p* < 0.05)	Yan et al.^28^ (Meta‐analysis of 18 studies)
Obsessive–compulsive disorder	483	DLPFC (11 studies) OFC (2 studies) SMA (2 studies)	Low to high frequency (1–20 Hz)	Improvement in OCD symptoms	2.94 (*p* = 0.002)	Trevizol et al.^37^ (Meta‐analysis of 15 studies)
	791	DLPFC (16 studies) OFC (2 studies) SMA (2 studies)	Low frequency (1 Hz) High frequency (5–20 Hz)	Improvement in OCD symptoms	0.73 (*p* < 0.001) 0.70 (*p* < 0.001)	Zhou et al.^38^ (Meta‐analysis of 20 studies)
Schizophrenia	827	DLPFC	Low to high frequency (1–20 Hz)	Improvement in negative symptoms	0.64 (*p* < 0.0001)	Aleman et al.^43^ (Meta‐analysis of 19 studies)
	768	DLPFC	Low to high frequency (1–20 Hz)	Improvement in hallucinations and negative symptoms	0.49 (*p* < 0.001)	Kennedy et al.^44^ (Meta‐analysis of 30 studies)
Suicide	593	DLPFC (9 studies) PFC (2 studies)	Low to high frequency (1–50 Hz)	Significant decrease in suicidal thoughts	ND	Bozzay et al.^53^ (Systematic review of 11 clinical trials)
	946	DLPFC	Low to high frequency (1–50 Hz)	Significant decrease in suicidal thoughts	ND	Serafini et al.^51^ (Systematic review of 16 studies)
Cognitive and memory deficits	293	DLPFC (9 studies) IFG (2 study) Temporoparietal regions (2 studies)	Low to high frequency (1–20 Hz)	Cognitive improvement in patients with MCI or Alzheimer's disease	0.91 (*p* < 0.0001) MCI 0.75 (*p* < 0.0001) Alzheimer's Disease	Chou et al.^57^ (Meta‐analysis of 13 studies)
	389	DLPFC (11 studies) IFG (1 study) Temporoparietal regions (3 studies)	Low to high frequency (1–20 Hz)	Improvement in memory, language, and executive functions in patients with MCI and Alzheimer's disease	0.45 (*p* = 0.01)	Wang et al.^59^ (Meta‐analysis of 15 studies)

Abbreviations: DLPFC, dorsolateral prefrontal cortex; IFG, Inferior frontal gyrus; MCI, Mild cognitive impairment; ND, not determined; OFC, orbitofrontal cortex; PFC, prefrontal cortex; SMA, supplementary motor area; SMD, standardized mean difference.

#### 2.1.1 Major depressive disorder

Patients with depression show a decreased activity in DLPFC area,^15^ and it has been shown that a stimulation with rTMS restores the functional connectivity of this area^16^ and relieves patients from depression.^17^ One of the first studies that reported the relief in depressive symptoms was through the application of high‐frequency (10 Hz) rTMS over DLPFC.^18^ Moreover, several meta‐analysis studies have shown that low‐frequency and high‐frequency rTMS applied to the DLPFC have antidepressant effects.^17^, ^19^, ^20^, ^21^ Furthermore, rTMS has also been applied in conjunction with cognitive behavioral therapy specifically in patients with drug‐resistant depressive disorder. The first known case study was conducted with a woman who received high‐frequency (10 Hz) rTMS for 14 weeks together with cognitive behavioral therapy and showed a reduction in depressive symptoms.^22^ Furthermore, a study combining self‐system therapy, which is similar to behavioral therapy, and high‐frequency (10 Hz) rTMS on the left DLPFC improved the severity of depression.^23^

#### 2.1.2 Anxiety disorder

Generalized anxiety disorder is a psychiatric disorder characterized by excessive and uncontrollable worry.^24^ Anxiety disorder is associated with abnormalities in the connectivity of frontal regions of the brain^25^ and a study in ten adult patients who received low‐frequency (1 Hz) rTMS on the right DLPFC for 3 weeks found an improvement of more than 50% in anxiety, and posttreatment remission rate reached to 60%.^26^ Similarly, another study with the application of high‐frequency (20 Hz) rTMS found significant improvement in the severity of anxiety.^27^ In addition, multiple systematic reviews and meta‐analysis studies showed that the application of rTMS ameliorated anxiety symptoms, which remained stable for long‐term.^28^, ^29^, ^30^, ^31^ The application of rTMS has also helped patients with comorbid anxiety.^32^, ^33^

#### 2.1.3 Post‐traumatic stress disorder

Post‐traumatic stress disorder (PTSD) is characterized by the appearance of symptoms following exposure to a stressful and traumatic event.^24^ Imaging studies showed that medial prefrontal cortex (mPFC) is hypoactive whereas amygdala is hyperactivated in PTSD patients^34^ and, compared with healthy controls, PTSD patients show poorer mPFC‐mediated regulation of the amygdala.^35^ Therefore, it has been argued that increased mPFC regulation of amygdala might mitigate the fear response and consequently facilitate extinction of the fear.^36^ In a double‐blind study with 30 patients, application of high‐frequency (20 Hz) rTMS bilaterally on the mPFC in 12 sessions significantly reduced PTSD symptoms.^35^ Another study applied rTMS on the right and left DLPFC to effectively reduce the symptoms of PTSD in most patients.^37^ In addition, several systematic reviews and meta‐analysis studies found that rTMS treatment effectively ameliorated the severity of PTSD symptoms.^28^, ^38^, ^39^, ^40^, ^41^ Furthermore, it has been suggested that the combination with exposure therapy by exposing to traumatic images before rTMS stimulation is particularly effective in treating patients with PTSD.^35^

#### 2.1.4 Obsessive–compulsive disorder

Obsessive–compulsive disorder (OCD) is a disorder with lifetime prevalence of 2–3%. The first approved noninvasive brain stimulation technique for the treatment of OCD in the European Union was deep TMS (dTMS), which allows stimulation of deeper subcortical structures and larger brain volume than conventional rTMS. Considering that orbitofrontal‐subcortical circuits underlie the symptoms of OCD, stimulation of orbitofrontal cortex by the application of TMS could in fact modulate this circuit activity and relieve OCD symptoms in patients.^42^, ^43^ In recent years, the application of high‐frequency dTMS has been shown to alleviate the OCD symptoms even in medication‐resistant patients.^44^, ^45^, ^46^ However, in the same line, several systematic reviews and meta‐analysis studies found that rTMS is also beneficial for the treatment of OCD symptoms.^47^, ^48^, ^49^ An application of rTMS in OCD patients caused significant improvement in symptoms.

#### 2.1.5 Schizophrenia

The psychopathological symptoms of schizophrenia are clustered in three main categories: cognitive, negative, and positive. Cognitive decline is a central feature of schizophrenia, and its improvement has been considered as a better predictor of recovery from schizophrenia than positive or negative symptoms.^50^ Meta‐analysis studies of psychiatric patients found that administration of rTMS over DLPFC caused no cognitive improvements ^51^ and produced no effect on working memory.^52^ By contrast, other meta‐analysis studies showed that the application of rTMS over the frontal cortex of schizophrenia patients induced significant improvement in the negative symptoms,^53^, ^54^ and a systematic review of 41 studies with a total of 1473 schizophrenics and a randomized clinical trial study with 27 schizophrenia patients found that rTMS improved auditory hallucinations and positive symptoms.^55^, ^56^ However, a systematic review concluded that only 12 of 30 studies included in the review found evidence of the beneficial effect of rTMS on positive symptoms in schizophrenia patients.^57^

#### 2.1.6 Suicide

Suicide has become a global public health problem mostly in developed countries.^58^ A study showed that after the application of high‐frequency (10 Hz) rTMS three times a day for 3 days in the left prefrontal cortex, none of the patients committed suicide in 6 months and their suicidal thoughts decreased.^59^ Similarly, other studies have revealed that an application of high‐frequency (5 or 10 Hz) rTMS on the frontal cortex significantly decreased suicidal thoughts in patients.^60^, ^61^, ^62^ In the same line, a systematic review of six randomized controlled trials and five unblinded trials with a total of 593 patients found that rTMS was beneficial for suicide patients.^63^ Additionally, application of high‐frequency (10 Hz) rTMS on the right DLPFC in 10 borderline personality disorder patients, which are a group of patients with high risk of suicide attempts, significantly improved affective instability and anger.^64^

#### 2.1.7 Cognitive and memory deficits

Cognitive and memory deficits are not only accompany normal aging but also comorbid with many brain diseases. A systematic review found that eight of 12 studies using rTMS application over DLPFC in elderly people showed significant cognitive improvements,^65^ and a meta‐analysis study concluded that rTMS has positive effect on cognitive improvement in healthy aging and Alzheimer's disease.^66^ In addition, numerous meta‐analysis studies have shown that the application of rTMS produces precognitive effects in patients with mild cognitive impairment and Alzheimer's disease^67^, ^68^, ^69^, ^70^ and in patients with cognitive deficits and dementia.^71^, ^72^, ^73^ The beneficial effect of rTMS treatment has also been shown in improving episodic memory in cognitively deficient individuals^74^ and in reverting cognitive impairments in celiac disease patients.^75^

### 2.2 Transcranial direct current stimulation

tDCS modulates cortical excitability by delivering electrical current through anodal and cathodal electrodes to facilitate or inhibit neuronal activity.^76^ A tDCS stimulation changes synaptic transmission,^77^, ^78^, ^79^ induces synaptic plasticity^80^ and metaplasticity,^14^ and extends to other brain areas through decrease/increase in axonal release of monoamine neurotransmitters, such as dopamine.^81^ tDCS has widely been used to treat several mental, neuropsychiatric, and cognitive disorders (see a summary in Table 2). In following, we describe on the therapeutic benefits of tDCS:

**TABLE 2:** Therapeutic benefits of application of tDCS in mental, psychiatric, and cognitive disorders

Disorder	Participant size	Stimulation site	Stimulus current density	Outcome of treatment	Effect size or SMD and *p*‐value	References and comment
Major depressive disorder	623	DLPFC	0.04–0.08 mA/cm^2^	Reduction in depression	3.95–5.20 (*p* < 0.00001)	Wang^71^ (Meta‐analysis of 9 studies)
	1092	DLPFC	0.028–0.10 mA/cm^2^	Reduction in depressive episodes	0.45 (*p* < 0.05)	Razza et al.^72^ (Meta‐analysis of 23 studies)
Anxiety disorder	349	DLPFC (8 studies) VLPFC (1 study) PFC (1 study) Sensory cortex (1 study)	0.06–0.08 mA/cm^2^	Significant reduction in anxiety symptoms	ND	Stein et al.^83^ (Review of 11 studies)
	37	DLPFC	0.06–0.08 mA/cm^2^	Significant reduction in symptom severity	ND	Vicario et al.^20^ (Systematic review of 5 studies)
	378	DLPFC	0.01–0.08 mA/cm^2^	Significant reduction in anxiety symptoms	0.67 (*p* < 0.001)	Cheng et al.^82^ (Meta‐analysis of 11 studies)
Post‐traumatic stress disorder	90	PFC (4 studies) Temporal cortex (1 study)	0.028–0.57 mA/cm^2^	Significant improvement in PTSD symptoms	ND	Gouveia et al.^87^ (Review of 5 studies)
	40	DLPFC	0.57 mA/cm^2^	Effective reduction in severity of PTSD symptoms	3.86 (*p* = 0.001)	Ahmadizadeh et al.^86^ (Clinical trial)
Obsessive–compulsive disorder	77	DLPFC (2 studies) OFC (7 studies) SMA (3 studies)	0.06–0.36 mA/cm^2^	Significant reduction in OCD symptoms	ND	Brunelin et al.^90^ (Systematic review of 12 studies)
	110	DLPFC (4 studies) OFC (7 studies) SMA (3 studies) mPFC (1)	0.028–0.36 mA/cm^2^	Significant improvement in OCD symptoms	ND	D'Urso et al.^81^ (Review of 15 studies)
	21	OFC	0.06 mA/cm^2^	Significant reduction in OCD symptoms	5.26 (*p* = 0.03)	Bation et al.^89^ (Clinical trial)
	43	SMA	0.06 mA/cm^2^	Significant reduction in OCD symptoms	0.62 (*p* = 0.03)	Silva et al.^91^ (Clinical trial)
Schizophrenia	338	Fronto‐temporoparietal areas (6 studies) DLPFC (4 studies)	0.06–0.08 mA/cm^2^	Improvement in negative symptoms and auditory hallucinations	0.41–1.04 (*p* < 0.04)	Kim et al.^92^ (Meta‐analysis of 10 studies)
	447	DLPFC	0.06–0.39 mA/cm^2^	Improvement in negative symptoms	0.31 (*p* < 0.05)	Yu et al.^94^ (Meta‐analysis of 14 studies)
	270	DLPFC	0.03–0.08 mA/cm^2^	Improvement in working memory	0.49 (*p* = 0.004)	Narita et al.^97^ (Meta‐analysis of 9 studies)
Cognitive and memory deficits	566	DLPFC (12 studies) VLPFC (1 study) Temporoparietal areas (9 studies) IFG (2 studies)	0.024–0.182 mA/cm^2^	Improvement in episodic memory in elderly individuals	0.40 (*p* = 0.002)	Huo et al.^110^ (Meta‐analysis of 24 studies)
	146	DLPFC (4 studies) Temporoparietal areas (3 studies)	0.06–0.08 mA/cm^2^	Improvement in cognitive functions in patients with Alzheimer's disease	0.37 (*p* = 0.01)	Cai et al.^105^ (Meta‐analysis of 7 studies)
	532	DLPFC (9 studies) VLPFC (1 study) PFC (5 studies)	0.027–0.06 mA/cm^2^	Improvement in cognitive functions and episodic memory in elder individuals	0.86 (*p* < 0.05)	Indahlastari et al.^104^ (Meta‐analysis of 13 studies)

Abbreviations: DLPFC, dorsolateral prefrontal cortex; IFG, Inferior frontal gyrus; mPFC, medial prefrontal cortex; ND, not determined; OFC, orbitofrontal cortex; PFC, prefrontal cortex; SMA, supplementary motor area; SMD, standardized mean difference; VLPFC, ventrolateral prefrontal cortex.

#### 2.2.1 Major depressive disorder

Several meta‐analysis studies suggest that the application of tDCS has antidepressant effects^82^, ^83^, ^84^ and this effect can last for long‐term.^85^ In addition, an application of tDCS combined with either antidepressant medications or psychotherapy was more effective treatment for depression than tDCS alone.^86^, ^87^, ^88^ The effectiveness of behavioral therapy in combination with tDCS was also observed in relieving patients from depression.^89^, ^90^

#### 2.2.2 Anxiety disorder

It has been shown that persons with anxiety tend to pay more attention to threaten signals than to neutral stimuli and that their left DLPFC is underactive during attention bias tasks.^91^, ^92^ Therefore, it was suggested that the stimulation of left DLPFC by the application of tDCS could improve the engagement of this brain area and rescue patients from anxiety.^91^ Systematic reviews and meta‐analysis studies found that the application of tDCS over DLPFC significantly reduced the anxiety symptoms.^30^, ^93^, ^94^ Furthermore, outcome from studies suggests that tDCS is more effective in reducing anxiety symptoms when used in combination with medications or cognitive behavioral therapy.^95^

#### 2.2.3 . Post‐traumatic stress disorder

A study in 28 war veterans suffering from PTSD found that the application of tDCS on prefrontal cortex caused improvement in PTSD manifestations.^96^ Another study from the same group showed that the exposure of war veterans with PTSD to combat‐related virtual reality during the application of tDCS on prefrontal cortex induced a considerable reduction in PTSD symptoms.^97^ Similar to these studies, systematic reviews and clinical trial studies have showed the efficacy of tDCS in reducing PTSD symptoms.^93^, ^98^, ^99^ Furthermore, PTSD patients subjected to a combination of working memory training and tDCS application showed significant improvements in a variety of parameters of cognitive and emotional behaviors.^100^

#### 2.2.4 Obsessive–compulsive disorder

A review of 15 studies found that the application of tDCS over either DLPFC, orbitofrontal cortex or supplementary motor area in patients with OCD induced significant reduction in symptoms.^93^ Similarly, the effectiveness of tDCS in reducing OCD symptoms has also been demonstrated in patients.^101^, ^102^, ^103^ Thus, tDCS is seen as a promising mode of therapy for the treatment of OCD; however, more studies involving neuroimaging and neurophysiological markers are needed to help identify the best treatment parameters.

#### 2.2.5 Schizophrenia

Several meta‐analysis studies found that tDCS significantly decreased auditory hallucinations as well as negative and positive symptoms in patients with schizophrenia.^53^, ^54^, ^104^, ^105^, ^106^, ^107^ By contrast, clinical trial and meta‐analysis studies in schizophrenics showed that the application of tDCS improved working memory performance; however, no improvement was observed in other cognitive domains.^108^, ^109^, ^110^

#### 2.2.6 Cognitive and memory deficits

A treatment with tDCS improves age‐related alterations in memory performance^111^ and reduces reaction time in cognition‐related tasks.^112^ Systematic reviews and meta‐analysis studies have shown that treatment with tDCS in elderly people caused slowdown in the progression of cognitive deterioration^113^ and improved performance across the wide range of cognitive and memory tasks.^114^, ^115^, ^116^ In addition, the beneficial effect of tDCS treatment on cognitive performance has also been demonstrated in patients with mild cognitive impairment and Alzheimer's disease.^69^, ^117^ Furthermore, stimulation with tDCS in aging individuals improved performance on semantic word generation task,^111^ naming,^118^ associative learning task,^119^ and delayed episodic memory tasks.^120^, ^121^, ^122^

## 3 INVASIVE BRAIN STIMULATION

The most common invasive techniques are deep brain stimulation (DBS) and vagus nerve stimulation (VNS). However, we will focus here on DBS because it is the most widely used method.

### 3.1 Deep brain stimulation

DBS treatment implies passing electric currents into the subcortical nuclei of the brain through surgically implanted electrodes. DBS delivers continuous stimulation into a targeted brain nuclei and changes its activity in a controlled way. DBS has frequently been used to help patients who show resistance to other viable therapies, such as prescription drugs, psychotherapy, or noninvasive stimulation therapy. Since the emergence of DBS as a therapy for patients with involuntary movement disorders in the late 1980s, it has been used for the treatment for several mental and psychiatric disorders (see a summary in Table 3). In following, we describe on the therapeutic benefits of DBS:

**TABLE 3:** Therapeutic benefits of application of DBS in mental and psychiatric disorders

Disorder	Participant size	Stimulation site	Stimulus	Outcome of treatment	Effect size, SMD or overall effect and p‐value	References and comment
Major depressive disorder	233	SCG (8 studies) VC/VS (2 studies) EPC (2 studies) MFB (2 studies) ALIC, PGR and NAC (1 study each)	60–180 Hz	Decrease in depressive symptoms	0.56 (*p* < 0.0001)	Wu et al.^120^ (Meta‐analysis of 17 studies)
	263	SCG (10 studies) VC/VS (3 studies) ALIC (2 studies) NAC (2 studies) MFB (2 studies)	60–180 Hz	Reduction in depressive symptoms	1.78 (*p* < 0.05)	McGirr and Berlim^119^ (Meta‐analysis of 19 studies)
Obsessive–compulsive disorder	310	VC/VS (7 studies) ALIC (6 studies) ALIC/NAC (6 studies) NAC (6 studies) NAC/BST (2 studies) ITP (4 studies) STN (6 study) Other areas (9 studies)	85–280 Hz	Reduction in OCD symptoms	4.41 (*p* < 0.0001)	Martinho et al.^129^ (Meta‐analysis of 46 studies)
	253	VC/VS (6 studies) ALIC (4 studies) NAC/BST (2 studies) NAC (3 studies) ITP (2 studies) STN (1 study)	85–280 Hz	Reduction in OCD symptoms	1.64 (*p* < 0.001)	Hageman et al.^130^ (Meta‐analysis of 18 studies)
Addiction	33	NAC (13 studies) NAC/ALIC (1 study)	130–185 Hz	Significant reduction in craving and consumption (43–61%)	ND	Hassan et al.^133^ (Systematic review of 14 studies)
	25	NAC	130–185 Hz	Significant reduction in craving and consumption	ND	Coles et al.^134^ (Systematic review of 9 studies)

Abbreviations: ALIC, anterior limb of the internal capsule; EPC, epidural prefrontal cortex; MFB, medial forebrain bundle; NAC, nucleus accumbens; ND, not determined; SCG, subcallosal cingulate gyrus; SMD, standardized mean difference; STN, subthalamic nucleus; ITP, inferior thalamic peduncle; VC/VS, ventral capsule/ventral striatum.

#### 3.1.1 Major depressive disorder

A long‐term study in patients treated with DBS showed a decrease of more than 50% in the severity of depression.^123^ This decrease in depression was 53% after 3 months and it reached to 71% after 18 months. Various brain areas have been identified for the application of DBS, including the cingulate cortex,^124^ the capsula interna,^125^ the nucleus accumbens,^126^ and the superolateral medial forebrain.^127^ A review of effect of DBS application in different brain areas from 22 studies found 40–70% improvement in depression.^128^ Furthermore, a study reported that after 2 years of chronic stimulation, response and remission rates were 92% and 58%, respectively, and none of the patients displayed moderate or severe depressive episode afterward.^129^ In the same line, several meta‐analysis studies found that a treatment with DBS in drug‐resistant depressive patients caused a significant and long‐term improvement in depressive symptoms.^130^, ^131^, ^132^

#### 3.1.2 Post‐traumatic stress disorder

There is greater level of activity in the amygdala in response to trauma‐related negative stimuli in PTSD patients compared with control subjects.^34^, ^133^ The intensity of the amygdala activity observed in the fMRI images correlates well with the severity of the symptoms.^134^, ^135^ Therefore, an intervention in amygdala is expected to benefit the PTSD patients. A study with a war veteran suffering with PTSD showed that DBS‐mediated stimulation of the basolateral nucleus of the amygdala reduced more than 37% the symptoms.^136^ Similar results were obtained in a follow‐up study of same patient and another PTSD patient.^137^ Another case study showed that DBS in medial prefrontal cortex/uncinate fasciculate caused full recovery of patient from PTSD symptoms.^138^ Beside these, there is no other study available in the literature on DBS treatment in PTSD patients.

#### 3.1.3 Obsessive–compulsive disorder

DBS was approved for OCD treatment by the Food and Drug Administration in 2009 for patients not responding to other treatments. A systematic review and meta‐analysis of 31 studies in treatment‐resistant OCD patients where 83 subjects received stimulation in striatal areas consisting of striatum, nucleus accumbens and caudate and 27 subjects in subthalamic nucleus and six subjects in inferior thalamic peduncle, found that DBS‐mediated stimulation in anyone of these brain target areas caused 45% reduction in the symptoms of OCD.^139^ Furthermore, in contrast to adult patients, a meta‐analysis of 21 studies with 58 children and youth ages between 12 and 21 years showed 57% improvement in OCD symptoms after stimulation with DBS.^140^ In the same line, several meta‐analysis studies showed that DBS is effective in reducing OCD symptoms in drug treatment‐resistant patients.^141^, ^142^

#### 3.1.4 Addiction

A case study revealed complete remission of heroin abuse in one male for 6 years after stimulation with DBS and patient remained abstinent even after turning off the stimulation.^143^ Similarly, another case report showed that stimulation with DBS in the nucleus accumbens caused prolonged cessation of heroin use.^144^ Furthermore, a systematic review of 14 studies found that the DBS in nucleus accumbens caused remission from 6 months to more than 6 years at a rate between 43 and 61%.^145^ Systematic reviews on the efficacy of DBS in multiple types of addiction revealed that nucleus accumbens stimulation with DBS induces long‐term abstinence in addiction‐related behavior.^146^, ^147^

## 4 CONCLUDING REMARKS

In this review, we have centered on the application of brain stimulation methods (rTMS, tDCS, and DBS) in successful treatment of mental, psychiatric, and cognitive disorders. After going through the literature, we found that both noninvasive and invasive methods offer a great potential in remedy against wide range of brain disorders not only for patients in general but also for patients who have shown resistance to medications and other therapies. It is no wonder why brain stimulation has become a preferred approach in the last decade in combating against brain disorders. However, the success of brain stimulation lies in the recent technological advancements that have evolved into the development of precise devices with capacity to produce well‐controlled and effective brain stimulation. Within the currently available noninvasive methods, rTMS is considered as a tool with great therapeutic potential because it is powerful and safe, and the risk of severe negative side effects upon application is very low.^148^ By contrast, tDCS is not as powerful and generates weak stimulus; however, it is relatively easy to use and transport, lot less expensive, and it has low incidence of side effects.^148^ Even though the tDCS is weak and it cannot induce high‐frequency stimulation, the use of this technique has increased 4.5 times in a decade compared with 1.5 times for rTMS according to the publications recorded in PubMed. Nevertheless, the rTMS remains the most widely used noninvasive technique. In contrast to rTMS and tDCS, DBS is often used as a last resort for treating patients who have shown no relief after other viable therapies. The advantage of DBS over noninvasive techniques is that DBS can selectively and precisely activate a set of nuclei deep in the brain and modulate their functions. However, compared with tDCS and TMS, DBS treatment in some of the brain nuclei has been shown to produce severe side effects. It has been observed that there is high rate of suicide in patients treated with DBS, particularly with stimulation in STN and GPi areas of brain. However, relatively low rates of depression, mania, cognitive impairment, and emotional and behavioral changes were reported.^149^ Within TMS, tDCS, and DBS techniques of brain stimulation, TMS is the most used in clinical applications and currently, more than 1300 clinical trials are registered in clinicaltrials.gov for TMS. However, with more than 1100 registered clinical trials, current pattern suggests that tDCS is catching up rapidly.

### FUTURE PERSPECTIVES

Brain stimulation technology continues to evolve, and more versatile tools are expected to develop in near future. Recently, we have seen the development of closed‐loop stimulation system, which is a device that automatically adjusts the stimulation parameters according to the clinical state of patient in real time. Closed‐loop stimulation system consists of sensing module that assesses the feedback variable; control module that interpretes the variable and elaborates the new stimulation parameters; and stimulation module that controls the delivery of stimulation. This closed‐loop system has been integrated into the DBS,^150^ which is called as adaptive DBS (aDBS). aDBS is a form of stimulation designed to overcome the technical limitations of typical DBS that delivers continuous stimulation to the target brain area without considering the symptoms or status of the patient. So far, aDBS has been tested in several neurological conditions, and currently, is under extensive study to translate it into clinical practice. The adaptation of closed‐loop system to TMS and tDCS has been used in some studies but it remains in developmental stage. Nevertheless, in parallel, many forms of rTMS have been developed over the time. Apart from the theta‐burst stimulation (TBS) and magnetic seizure therapy (MST), some of the newer forms are accelerated rTMS (aTMS), priming TMS (pTMS), and synchronized TMS (sTMS). These forms differ from each other in the application of frequency of magnetic pulses. More recently, Stanford Accelerated Intelligent Neuromodulation Therapy (SAINT), a kind of accelerated rTMS, has been approved by FDA for the treatment of depression. Additionally, deep TMS coils are also taking a center stage in brain stimulation because in contrast to standard coils, which are used to stimulate relatively superficial locations on the cortex, they permit penetratration of electromagnetic pulses deep into the brain. However, the caveat is that deep TMS coils target broader brain areas and cannot be used for the modulation of a specific area. Therefore, although deep TMS coils are very promising alternative to invasive brain stimulation methods, they remain limited and in future, it is desirable that deep TMS coils may adapt in a device that not only can penetrate deep into the brain but also can focally modulate a specific region and only that region.

## FUNDING INFORMATION

Research, Development, and Innovation Plan of Junta de Andalucía in Spain, group grant/award number: CTS 586/21.

## CONFLICT OF INTEREST

Authors declare no conflict of interest.

## Data Availability

Considering that this is a review article, data sharing is not applicable.
